# A Vexing Problem of Air Trapping in the Trima Accel During Plateletpheresis Procedure: Reflecting Our Experience in Troubleshooting

**DOI:** 10.7759/cureus.38518

**Published:** 2023-05-03

**Authors:** Suman S Routray, Sukanta Tripathy, Gopal Ray

**Affiliations:** 1 Immunohaematology and Blood Transfusion, Kalinga Institute of Medical Sciences, Bhubaneswar, IND; 2 Transfusion Medicine, All India Institute of Medical Sciences, Guwahati, IND

**Keywords:** troubleshooting, fishbone analysis, apheresis, air block, access pressure

## Abstract

Troubleshooting for any fault of apheresis equipment or kit is hardly addressed. Here, we report a unique problem of air trapping in a kit at two different positions leading to failure of the plateletpheresis procedure. Two plateletpheresis procedures were aborted due to “Access pressure low”, though the needle position was absolutely perfect. In the third event, platelet yield was not increasing even after 30 minutes from the time of initiation. It was completed after stopping the centrifuge pump which could have displaced the air bubble from the collection port. The root cause for these events was analyzed in consultation with the apheresis technical expert and “air block” was found to be the cause. Air block can also result in a “low access pressure” alarm despite improper phlebotomy being the common cause. Perfect kit loading, checking of tubing defects prior to loading, and comparative analysis of troubleshooting to have adequate knowledge are essential tools for the smooth functioning of apheresis.

## Introduction

Platelet transfusion is used therapeutically to treat bleeding patients or prophylactically to prevent bleeding. Platelets can be processed from whole blood donation (whole blood derived platelets) or by apheresis technology (single donor apheresis platelets, SDAP). The Trima Accel apheresis machine (version 6.0; Terumo Penpol Pvt Ltd., Thiruvananthapuram, India) has been the preferred equipment for plateletpheresis because of claims of better platelet yield, collection efficiency, collection rate, and low adverse donor reaction compared to other available apheresis equipment [1,2]. Troubles during the plateletpheresis procedure, either due to equipment or donor-related factors, can lead to the termination of the procedure and wastage of this scarce resource, resulting in higher production costs. Though donor-related adverse events are implicated in procedure abandonment, there is hardly any report on troubleshooting that addresses the faults associated with the plateletpheresis kit. Failure to detect leakage during the plateletpheresis procedure while using the Trima Accel system has been documented in the literature [3]. Here, we report a unique problem of air trapping in the kit, resulting in the termination of the procedure.

## Technical report

Procedure protocol followed in our centre

Donor selection for SDAP at our centre is done as per the standard guidelines elucidated in the Drug and Cosmetic Act, 1945 (amended March 2020) [4]. Following counseling and a medical examination of the donor, two blood samples in ethylenediaminetetraacetic acid (EDTA) vials are collected for blood counts, grouping, and the mandatory serology screening tests, respectively. A donor with a platelet count of more than 1.5 lakh/cmm, with the same blood group as the patient, non-reactive with serology screening, and fit as per the donor selection criteria is selected for SDAP donation. A written consent which includes the procedural details with anticipated adverse donor reactions and technical errors is obtained from the donor and the patient attendant. Oral chewable calcium phosphate IP (Ostocalcium plus; GlaxoSmithKline Pharma, Philadelphia, PA, USA) originally containing 500 mg of essential calcium is administered to the donor to alleviate any citrate-related side effects.

A single-use apheresis kit is installed in the machine as per the manufacturer's instructions. Acid citrate dextrose (ACD) is used as an anticoagulant to keep the line (access line to return line) patent. The Trima Accel equipment calculates the total blood volume of the donor after entering donor details, including weight, height, gender, blood group, hematocrit, and platelet count, into the software. Depending on the target yield desired, the device calculates the duration of the procedure. The pinch clamps are closed after the parameters have been verified, and the kit integrity testing is carried out using a pressure gradient system. Before attaching to the donor's cleaned arm, the circuit is primed with anticoagulant and alarm checks are made. In order to create an air-free circuit below the level of the blue blood diversion pinch clamp and to look for any leaks in the system, integrity testing is essential. Air is diverted to the vent bag if any air is found at this stage that is below the level of the blue blood diversion pinch clamp, which causes a vacuum in the system. Integrity testing, priming cycles, and alarm checks were carried out in our case in accordance with protocol, and everything went smoothly.

Event details

Event 1

After completing the protocol as described above, phlebotomy for the procedure was performed on the right cubital vein with a beveled up side needle. But though the phlebotomy was perfect and the needle was at the proper position, the blood flow halted just after the venipuncture needle without a single drop of blood slithering into the diversion pouch. All the troubleshooting steps for reduced access pressure were performed following the machine's instructions on the display screen. Despite all measures, the access line could not detect blood flow. We appreciated air in the diversion pouch, which on pressing pushed the blood column back. So the access line was changed to the other arm, to clear the air column from the diversion pouch. But the same trouble of "access pressure low'' persisted, and the procedure was aborted.

Event 2

After venipuncture in the left median cubital vein for the procedure, blood slithered to the diversion pouch, but with the closure of the diversion pouch and opening of the access line, the blood flow did not cross the junction of these two lines. The machine displayed the alarm “Access pressure low”. The tubing of the kit, needle position, and vein were inspected thoroughly, but no fault could be detected. The access line was made patent with draining few drops of anticoagulated blood and no clot was detected, then it was changed to the other arm. However, the same trouble of "access pressure low'' persisted, resulting in the abandonment of the procedure.

Event 3

Usually, the platelet collection in the collection bag starts 10 to 15 minutes after the commencement of the procedure. However, in this case, even after 30 minutes, the collection valve did not open. Following the manufacturer's instructions, the centrifuge pump was stopped and restarted again. The procedure was completed with a yield of 3.0 × 10^11^ with a time duration of 62 minutes.

**Figure 1:**
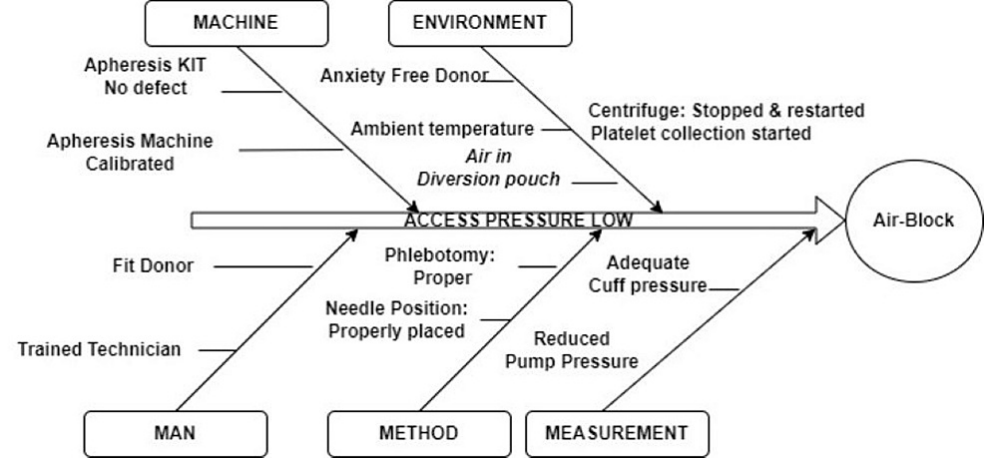
Fishbone analysis for the “Low access pressure” The root cause analysis of "access pressure low" using the fishbone tool by the Authors depicts that air block is due to the presence of air in the diversion pouch (first and second events) and in the inner channel (third event).

The root cause analysis (RCA) for these events was carried out in consultation with the apheresis technical expert (Terumo Penpol Pvt Ltd.). The details of the fishbone diagram used to identify many possible causes for the specific problem are depicted in Figure 1. The “air block” was detected as the root cause of the above events.

## Discussion

Plateletpheresis-related adverse events can be either due to donor reactions or technique-related or kit/equipment-related adverse events. Adverse donor reactions like vascular injuries, citrate toxicity, and vasovagal reactions are very few (0.7% to 6.06%) [5-8]. Adverse events arising from kit/equipment-related issues include hemolysis, air embolism, thrombosis, leakage, and infections. Though rare, these could be primarily because of manufacturing defects [9,10]. Low access pressure is a common fault seen during plateletpheresis, followed by spillover and non-increment of platelet yield [5].

In the first and second events, even after following the maneuvers mentioned above, the "draw pressure low" alarm was continuous, and ultimately, the procedure was aborted. The primary cause of this is incorrect phlebotomy, in which the vein was punctured excessively deeply or through. The vein's retained tonicity and the painless, smooth needle insertion in this case supported appropriate phlebotomy. The sudden stoppage of flow into the access line is primarily due to an air block, which can be cleared off by pushing normal saline through the designated port towards the diversion pouch or through the needle if the donor has been disconnected. If the sample bag pinch clamp at the diversion pouch is not properly closed during the priming cycle, air may become trapped in the pouch. Furthermore, if the diversion pouch is closed tightly without first letting the air out, the air cannot escape, resulting in entrapment. The technical experts after a thorough deliberation following receipt of many such similar problems from other users have assured to address such issues in the next upgraded version. They are planning to remove the needle line clamp from the new modified kit to allow the air (if present) to exit the needle line and generate a pressure test error alert instructing the operator to verify clamp closure (Appendix). Even though the current manufacturer's instructions state to open the white pinch clamps (both needle line and sample line) after phlebotomy, this air block can also be avoided by opening the clamps just before phlebotomy. Before phlebotomy, open the white pinch clamps just enough to let any air escape through the needle line. However, if these pinch clamps are opened following phlebotomy, the entrapped air in the diversion pouch or the tubing above the level of the blue blood diversion pinch clamp will be sucked into the vacuum circuit, resulting in a low-pressure alarm. The manufacturer's instructions address many other troubleshooting issues (Table 1) [11].

**Table 1:** Troubleshooting for the different types of alarms in Trima Accel BP: blood pressure, RBC: red blood cell, AC, anticoagulant, HCT: hematocrit

SN	Resolution steps for “Draw Pressure Low”	SN	Resolution steps for “Return Pressure High”
1	Ask the Donor to Squeeze the Hand grip	1	Ask the Donor not to Squeeze the Handgrip
2	Check the BP Cuff Pressure & Increase it up to 40 mm Hg	2	Look for the return line and the phlebotomy site. Is there any hematoma?
3	Check the Needle Position & adjust if required	3	Check the BP Cuff Pressure & reduce it if required
4	Reduce the Draw Pump Speed	4	Check the Needle Position & adjust if required
		5	Reduce the Return Pump Speed
	“Centrifuge Pressure High”		“Pressure Test Failure”
1	Stop the Centrifuge	1	Check if both the White Clamps in the donor line are closed
2	Open the Centrifuge Door & Check if the Kit is loaded properly & there are no kinks	2	Check if any tubing is stuck behind the Cassette or any Obstructions, if so, touch unload, clear the obstruction Obstruction & then Load again
3	Close the Door & Press Continue	3	Check if the Pump Headers & Cassette are loaded Correctly
	“Low Platelet Concentration Detected”		“Leak Detected”
1	Check for the Fluid in Plasma & Platelet line, if not present, stop the Centrifuge & then restart it	1	Stop the Centrifuge
		2	Open the Centrifuge Door
2	Check for Platelet Clumping & reduce the same (by increasing the AC ratio)	3	Check for any Leakage. If any leakage then aborts the procedure.
3	Increase the Platelet Yield	4	If any moisture, clean the Leak Detector and restart
		5	Touch Continue to Resume the Procedure
	“RBC Spillover”		“Level Sensor Error”
1	Increase the HCT by 3% and a maximum of up to 9%	1	Check for any air bubbles in the Reservoir Low-Level Sensor & TAP the Reservoir to clear it

In the third event, despite performing the procedure for 30 minutes, the platelet collection had not begun. The presence of air in the internal channels could be the cause of the product not being collected even though the system was running. The air bubble may be visualized in the platelet line or in the collection port, which prevents the flow of the platelets through the leukoreduction system (LRS) chamber to the collection port, diverting the platelets to the red blood cell (RBC) and plasma line, thus, returning back to the donor. This could be resolved by stopping the centrifuge pump or selecting the "air block" icon on the screen. When the "air block" icon is touched, a confirmation dialogue box with the adjustment or return to collection procedure is displayed. On choosing " adjustment", an automated system is initiated to clear the air block by reverse contribution, which pumps the air from the RBC line to the reservoir. The process is repeated till the air block is removed. The stopping-off centrifuge also has the ability to clear the block and provides the additional benefit of re-verifying the kit's correct loading. We stopped the centrifuge in our case, which moved the air bubble from the collection port to the RBC line and eventually to the reservoir. When the draw pressure is low, the problem is resolved by asking the donor to squeeze the hand grip, increasing the blood pressure (BP) cuff pressure to 40mmHg, while positioning the needle properly, and reducing the draw pump speed. Here, all these steps couldn’t resolve the glitch. The issue was resolved when the centrifuge was stopped, supporting the "air block" theory. The absence of specific alarms to detect any air in the inner channel hinders the identification of the actual cause.

## Conclusions

Air block can also result in a “low access pressure” alarm despite improper phlebotomy being the common cause. Additionally, ignorance of the air block can result in wasted kits, unnecessary changes in the access line, and increased donor anxiety about further donations. Safety aspects affecting the donor or the patient have the highest priority in any apheresis procedure. Perfect kit loading, checking for tubing defects prior to loading, and the right troubleshooting skills are essential for the apheresis procedure to run smoothly. A comparative analysis of troubleshooting during apheresis procedures using different machines with a large sample size will give us a more comprehensive view and help us solve this troublesome problem.

## Figures and Tables

**Figure 2:**
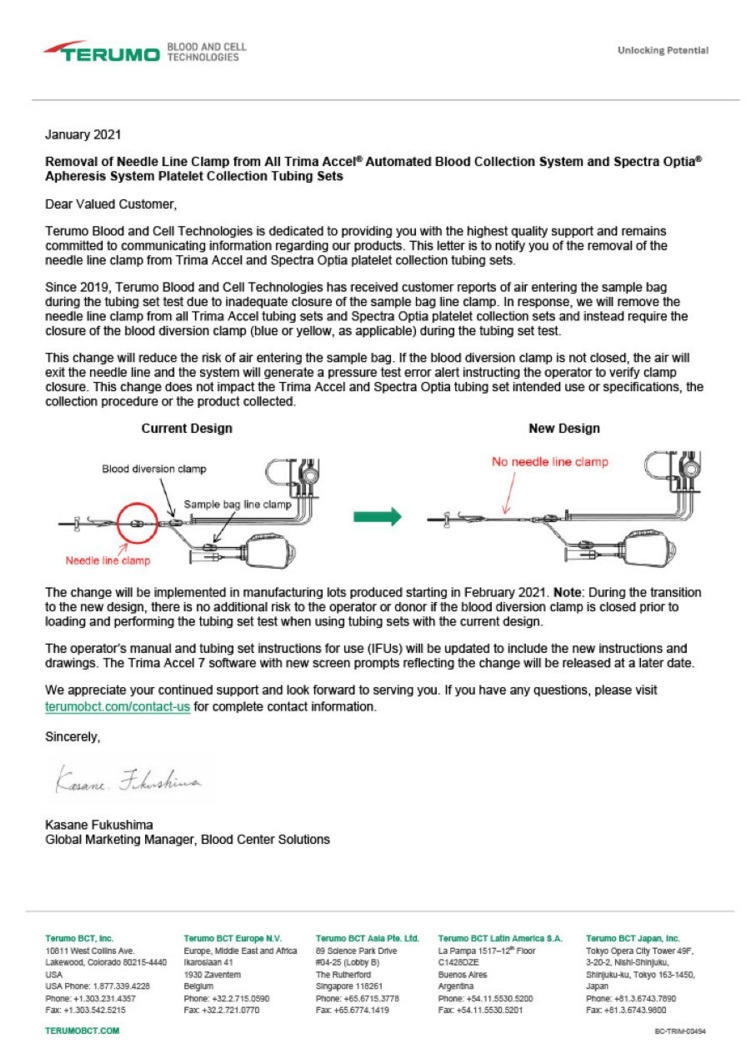
New needle line design

## References

[1] Arcot PJ, Kumar K, Coshic P, Andriyas V, Mehta V (2021). A comparative study of five plateletpheresis machines in a tertiary care center of India: AmiCORE vs COM.TEC vs Haemonetics MCS+ vs Spectra Optia vs Trima Accel. J Clin Apher.

[2] Keklik M, Korkmaz S, Kalan U, Sarikoc M, Keklik E (2016). Effectiveness of the Trima Accel cell separator in the double dose plateletpheresis. Transfus Apher Sci.

[3] Patidar G, Hans R, Mittal K, Malhotra S, Sharma RR, Marwaha N (2014). Failure of detection of leakage by cell separator during plateletpheresis procedures. Asian J Transfus Sci.

[4] (2023). Drug and Cosmetic act. March.

[5] Sahoo D, Mahapatra S, Parida P, Panigrahi R (2017). Various aspects of plateletpheresis: its impact on donor and patients. Glob J Transfus Med.

[6] Philip J, Sarkar RS, Pathak A (2013). Adverse events associated with apheresis procedures: incidence and relative frequency. Asian J Transfus Sci.

[7] Bassi R, Thakur KK, Bhardwaj K (2017). Plateletpheresis adverse events in relation to donor and plateletpheresis session profile. Iraqi J Hematol.

[8] Das SS, Sen S, Chakrabarty R (2018). Machine and man individualities in apheresis adverse events. Glob J Transfus Med.

[9] Mörtzell Henriksson M, Newman E, Witt V (2016). Adverse events in apheresis: an update of the WAA registry data. Transfus Apher Sci.

[10] Crookston KP, Novak DJ (2010). Physiology of apheresis. Apheresis: Principles and Practice.

[11] (2022). Trima Accel Service Manual - Frank's Hospital Workshop. Sep.

